# Minimal clinically important difference, substantial clinical benefit, and patient acceptable symptomatic state associated with upper extremity patient-reported outcome measurement information system scores following the Latarjet procedure

**DOI:** 10.1016/j.xrrt.2025.07.018

**Published:** 2025-08-13

**Authors:** Brian O. Molokwu, Jacquelyn J. Xu, Aidan G. Papalia, Paul V. Romeo, Matthew G. Alben, Hartej Singh, Mandeep S. Virk

**Affiliations:** Division of Shoulder and Elbow Surgery, Department of Orthopedic Surgery, NYU Grossman School of Medicine, NYU Langone Orthopedic Hospital, NYU Langone Health, New York, NY, USA

**Keywords:** Latarjet procedure, MCID, SCB, PASS, Shoulder Instability, Factors

## Abstract

**Background:**

The Patient-Reported Outcome Measurement Information System (PROMIS) has been widely used to assess clinical improvement in orthopedic procedures, providing a standardized and responsive measure of function and pain. While PROMIS has been effective in evaluating recovery in upper extremity surgery, specific thresholds for meaningful improvement following the Latarjet procedure (LP) have not been established. The purpose of this study was to determine the minimal clinically important difference (MCID), substantial clinical benefit (SCB), and patient acceptable symptomatic state (PASS) of PROMIS Upper Extremity (P-UE) Computer Adaptive Testing v2.0, Pain Interference (P-Interference), and Pain Intensity (P-Intensity) scores in patients undergoing the LP for shoulder instability. We hypothesize that PROMIS instruments will effectively distinguish these thresholds for clinically significant improvement with respect to shoulder function and pain following LP.

**Methods:**

MCID, SCB, and PASS were calculated using an anchor-based approach at a minimum follow-up of 1 year. The optimal cutoff values for change in PROMIS scores were determined via receiver operating characteristic curves and area under the curve analysis. Regression analysis was conducted to identify patient factors associated with achievement of the MCID, SCB, and PASS thresholds.

**Results:**

A total of 72 patients were included in our analysis. MCID for P-UE, P-Interference, and P-Intensity was determined to be 3.2, −6.3, and −9.4, respectively. Respective SCB values were determined to be 8.1, −10.7, and −11.4. Respective PASS values were determined to be 42.6, 56.7, and 39.4. Corresponding effect size and standardized response means were: P-UE (1.6, 3.4), P-Interference (1.4, 1.2), and P-Intensity (1.7, 1.2). For P-UE, generalized joint laxity was associated with lower odds of achieving SCB. For P-Interference, history of recurrent dislocations was linked to lower odds of achieving MCID. For P-Intensity, generalized joint laxity and history of recurrent dislocations were associated with lower odds of achieving MCID and SCB. Larger Hill-Sachs lesion depth was associated with lower odds of achieving P-intensity PASS.

**Conclusion:**

This study establishes MCID, SCB, and PASS values for PROMIS instruments following LP. Knowledge of these thresholds and patient factors associated with achieving them provide surgeons useful tools for predicting and measuring clinically meaningful outcomes following surgery.

Recurrent anterior shoulder instability is a common shoulder condition occurring primarily in active young males, especially in the setting of high-contact and high-fall-risk sports.^12^^,^^48^^,^^72^ The Latarjet procedure (LP) involves transfer of the coracoid process and conjoint tendon to the anteroinferior glenoid, providing both bony augmentation and dynamic stabilization to reduce the risk of recurrent instability. LP has a consistent track record of achieving excellent surgical outcomes for recurrent anterior shoulder instability with substantial glenoid bone loss (GBL).^33^^,^^55^ The LP demonstrates low rates of recurrent instability and excellent patient-reported outcomes measurements (PROMs).^53^^,^^55^^,^^56^ As utilization of LP continues to increase with expanding indications, it is increasingly important for surgeons to understand what PROMs constitute clinically meaningful outcomes.^54^

To improve clinical care and more efficiently collect PROMs, the National Institute of Health developed the Patient-Reported Outcomes Measurement Information System (PROMIS).^8^^,^^20^^,^^22^ Unlike traditional surveys, PROMIS involves the use of item-response theory and computer adaptive testing to reduce repetitive questions and lessen survey question burden.^8^^,^^20^^,^^22^ Since its inception, PROMIS has demonstrated its valuable clinical usability and has been validated across numerous orthopedic subspecialties.^3^^,^^8^^,^^20^^,^^22^^,^^24^^,^^28^^,^^32^^,^^63^ However, when clinically implementing PROMIS, it is necessary to differentiate between relevant clinical outcomes vs. only those that are statistically significant.^8^^,^^20^ In order to do this, the minimal clinically important difference (MCID), substantial clinical benefit (SCB), and patient acceptable symptomatic state (PASS) must be evaluated for the specific PROMIS instrument in use.

The purpose of this study was to establish the MCID, SCB, and PASS for PROMIS Upper Extremity (P-UE) Computer Adaptive Test Version 2.0 , Pain Interference (P-Interference), and Pain Intensity (P-Intensity) scores for patients undergoing LP. Moreover, our study sought to determine the responsiveness of these PROMIS instruments and what factors are associated with patients achieving these clinical thresholds. We hypothesize that PROMIS instruments will effectively distinguish these thresholds for clinically significant improvement with respect to shoulder function and pain.

## Materials and methods

### Ethics

Approval for this study was granted by the NYU Langone Health internal Institutional Review Board (S20-00287). All subjects provided informed consent prior to enrollment.

### Study design

This was a retrospective case series conducted on subjects from our institutional database between November 2018 and August 2024 using Current Procedural Terminology code 23462 (*capsulorrhaphy, anterior, any type; with coracoid process transfer*). All patients were treated by 1 of 4 fellowship-trained orthopedic surgeons, including 2 specializing in Shoulder and Elbow surgery and 2 in Sports Medicine. Inclusion criteria included patients who were at least 16 years of age at the time of the procedure and underwent LP for anterior shoulder instability with either critical unipolar or bipolar bone loss, or subcritical bone loss in the setting of additional risk factors such as participation in contact sports.^5^^,^^25^^,^^61^ Patients were required to have completed preoperative PROMIS surveys (P-UE, P-Interference, and P-Intensity) and have a 1-year minimum follow-up. Patients who were unable to provide informed consent or complete the surveys were excluded.

### Data collection

Patients who consented to participate were asked to complete 5 surveys. The first survey screened for prior surgery or traumatic injury to the shoulder operated on. The subsequent 3 surveys were P-UE, P-Interference, and P-Intensity, respectively. Anchor questions were included in the fifth and final survey to evaluate patients' perceived improvement with respect to shoulder function, pain, and overall satisfaction based on their current symptoms. Depending on patient preference, surveys were administered over the phone or sent via email. Of note, no significant differences in patient outcome scores have been shown when collecting data digitally or over the phone.^4^ Following survey completion, patient responses were stored securely within our institutional REDCap database (Vanderbilt University, Nashville, TN, USA).^21^

### Operative technique

Both open and arthroscopic LPs were performed with patients positioned in the beach-chair orientation using previously described standard techniques.^34^^,^^71^ Specifically, open deltopectoral approach with a subscapularis split was performed for the open technique. The coracoid was transferred to the anterior glenoid neck (3:00-6:00 position), with the lateral cortical surface of coracoid flush with the glenoid articular surface (classic Latarjet), and secured with 2 cortical screws (3.75 mm). The arthroscopic technique was performed as previously described by Lafosse et al.^34^

### Patient-reported outcome measures

All subjects completed the P-UE, P-Interference, and P-Intensity, Computer Adaptive Test version 2.0. PROMIS utilizes a T-score metric, normalized across a scale of 10 to 100, where the reference population mean is 50 with a standard deviation (SD) of 10.^19^^,^^20^^,^^22^ P-UE evaluates activities and motions requiring the use of the upper extremity, with higher scores correlating to increased functioning. P-Interference evaluates limitations of motion secondary to pain, while P-Intensity assesses the patient's level of subjective pain. Higher scores on P-Intensity and P-Interference, respectively, correlate with higher levels of pain and movement limitations secondary to pain.

### Anchor questions

Anchor questions utilizing a global rating of change scale were employed to assess physical function and pain levels of the postoperative shoulder. Patients were asked 2 anchor questions: “Compared to right before surgery, how would you describe the pain in your shoulder now?” (Anchor Pain) and “Compared to right before surgery, how would you describe the physical function of your shoulder now?” (Anchor Function).^20^ Patient responses were recorded using a 7-point Likert scale including: Much Better, Better, Slightly Better, No Change, Slightly Worse, Worse, and Much Worse. To determine PASS, patients were asked a third anchor question: “Taking into account all the activities you have during your daily life, your level of pain, and your functional impairment, how satisfied are you with your shoulder operation?” (Anchor Satisfaction). Responses were recorded on a 5-point Likert scale from: Very Satisfied, Satisfied, Neither Satisfied nor Dissatisfied, Dissatisfied, and Very Dissatisfied.

### Minimal clinically important difference, substantial clinical benefit, and patient acceptable symptomatic state, and responsiveness calculation

While MCID and SCB provide insight into the minimal and optimal thresholds for clinical improvement, respectively, PASS denotes the threshold at which a patient would consider themself well.^13^^,^^15^^,^^30^^,^^40^^,^^41^ The MCID and SCB were calculated using an anchor-based method via receiver operating characteristic analysis to determine the change in score that most accurately classifies patients reporting a specific transition rating on the anchor question.^20^ A response of “No change” was used as the reference standard. Of note, previous literature has demonstrated that the anchor-based approach for determining MCID, SCB, and PASS values is more clinically relevant than the distribution-based method.^27^ Anchor responses of “Much Better” were used for the SCB, while answers of “Slightly Better” were used for MCID as they reflected the ideal substantial and minimal improvement following the LP, respectively.^20^ Youden index determined the optimal cutoff point calculation for the maximum sensitivity and specificity; these were deemed to be acceptable if the area under curve (AUC) was greater than 0.7 and “excellent” if greater than 0.8.^20^, ^21^, ^22^^,^^47^

PASS was similarly calculated as the postoperative PROMIS score that most accurately reflects patients reporting the transition ratings of “very satisfied” or “satisfied.” These calculations are in accordance with similar studies in shoulder surgery, proximal interphalangeal joint arthroplasty, and shoulder arthroplasty.^20^^,^^22^^,^^43^^,^^67^ While the absolute change in PROMIS scores is necessary for MCID and SCB evaluation, PASS provides a PROMIS value beyond which patients would be satisfied with their outcome.^40^^,^^41^^,^^66^ This can, in turn, be utilized to classify subjects as “nonresponders” and “responders”.^65^ In addition, it can be employed by medical professionals to manage patient expectations and set treatment goals.^45^^,^^66^

Instrument responsiveness to change was calculated for each of the PROMIS instruments using the standardized response mean (SRM) and effect size (ES; Cohen d). ES is defined as the average difference between baseline and postoperative scores divided by the SD of both measurements.^65^ SRM is determined by dividing the average difference between baseline and postoperative scores by the SD of the difference between paired measurements.^46^ Confidence intervals (CIs) for the ES and SRM were estimated using bootstrap resampling (5,000 iterations). Absolute values of each measurement were defined as small (0.2), medium (0.5), or large (0.8).^37^^,^^46^^,^^65^

### Statistical analysis

Generation of receiver operating characteristic curves and tables, along with univariate and multivariate logistic regression analyses were performed using RStudio version 4.0.3 (Posit, Boston, MA, USA). To determine the potential predictors of achievement of PASS, MCID, and SCB based on the identified cutoff values, a univariate logistic regression was conducted for numerous patient variables. Variables that achieved a *P* value of .20 or less were then included in a multivariate logistic regression analysis. Ultimately, variables that achieved a *P* value below .05 in the multivariate analysis were deemed statistically significant, and odds ratios (ORs) were calculated for these significant variables. This methodology has been utilized in previous studies assessing clinically significant thresholds.^2^^,^^70^ A priori power analysis was performed to ensure sufficient power for the overall model given the available sample size. Factors considered for the logistic regression analysis included male sex (assigned at birth), primary vs. revision LP, age, body mass index (BMI), competitive athlete status, American Society of Anesthesiologists class, smoking status, GBL, Hill-Sachs lesion depth (mm), on-track versus off-track Hill-Sachs lesion, anxiety, history of depression, preoperative visual analog scale, preoperative PROMIS scores, active preoperative range of motion (abduction and external rotation), presence of preoperative generalized joint laxity (via Beighton score),^42^ history of recurrent dislocations, history of traumatic injury to shoulder, diabetes mellitus, hypertension, open vs. arthroscopic LP, and dominant arm procedure. Active shoulder flexion, extension, and internal rotation were excluded from the analysis because of documentation inconsistencies. A revision LP was defined as an LP that was performed following a failed arthroscopic Bankart repair (ABR). Competitive athlete status was defined as participation in organized sports at the high school level or higher, with regular practice or competition within 12 months prior to surgery. History of recurrent dislocations was defined as more than 1 dislocation event prior to the initial stabilization surgery (ie, prior to ABR in the context of a revision LP). Preoperative computed tomography data were available for all included patients. Radiographic factors, including bipolar bone loss, were assessed using preoperative computed tomography and/or magnetic resonance imaging as described^16^^,^^60^; while remaining patient factors were collected retrospectively from the electronic medical record.

## Results

### Demographics and outcomes scores

**Fig. 1:**
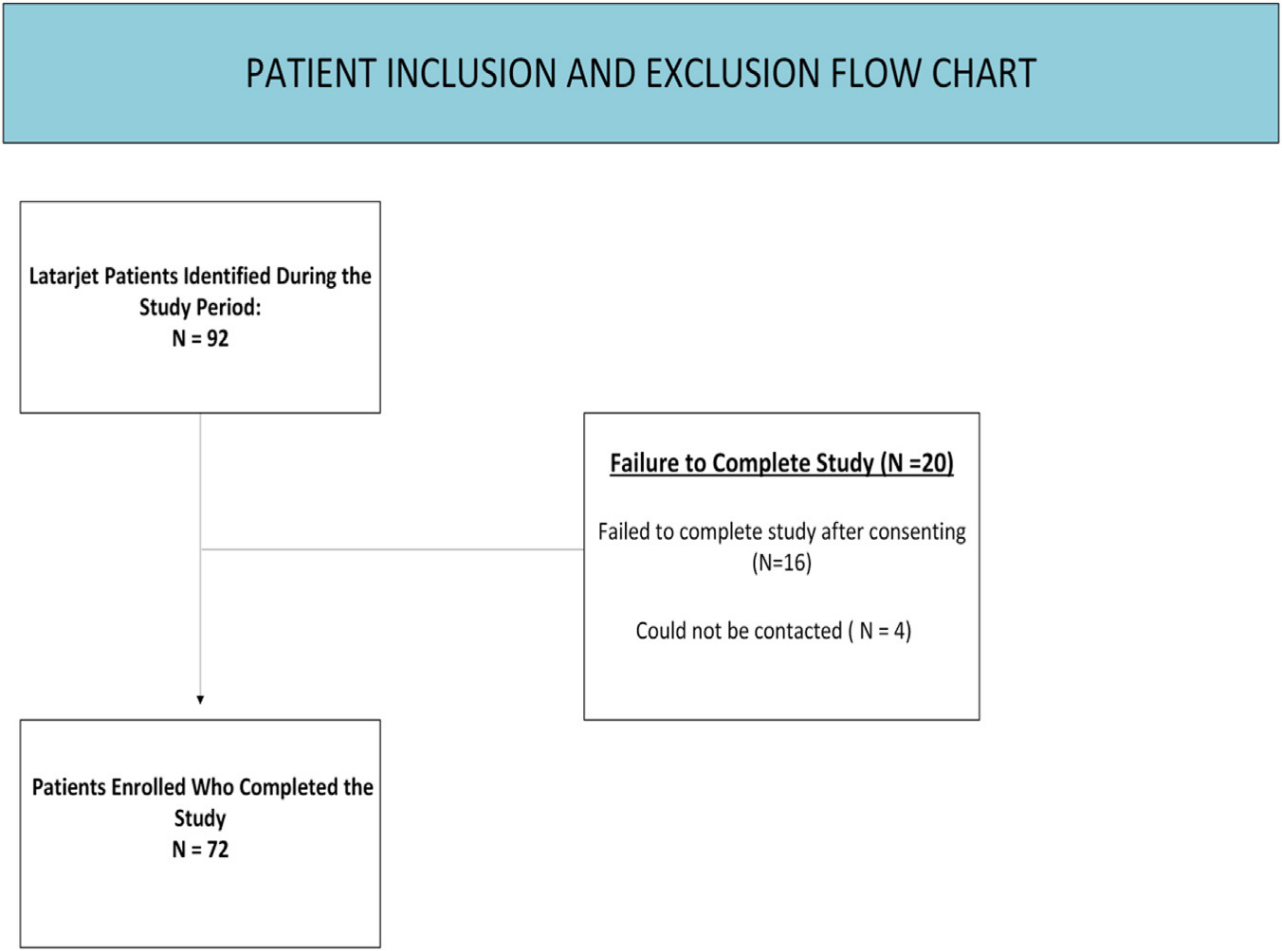
Patient inclusion and exclusion flowchart.

We identified 72 patients (64 male, 8 female) who met the inclusion criteria for this study out of a cohort of 92 total patients (Figure 1). The average age at surgery was 28.8 years (range: 16-59). Comprehensive patient demographics can be seen in Table I. Sixty patients underwent an open LP, while 12 underwent an arthroscopic LP. No concomitant procedures, such as remplissage, were performed in the patients who underwent the arthroscopic LP. Complete PROMIS scores were collected for all 72 patients (50 responded via email, 22 responded via telephone). There was 1 postoperative dislocation (1.4%), but no patients required reoperation, and no other complications were observed. Average preoperative scores for the full cohort included a P-UE of 38.2 ± 9.5, P-Interference of 57.4 ± 7.4, and P-Intensity of 46.7 ± 9.1. At average follow-up of 24 (range: 12-64) months, the average postoperative scores included a P-UE of 51.8 ± 10.1, P-Interference of 44.1 ± 9.8, and P-Intensity of 35.7 ± 7.9 for the full cohort. Preoperative and postoperative scores were comparable between patients undergoing primary and revision LP and between open and arthroscopic techniques (Table II). The distribution of patient responses to the 3 anchor surveys are demonstrated in Table III. Anchor-based MCID for P-UE, P-Interference, and P-Intensity were 3.2 (AUC 0.88), −6.3 (AUC 0.86), and −9.4 (AUC 0.84), respectively. PASS for P-UE, P-Interference, and P-Intensity were 42.6 (AUC 0.92), 56.7 (AUC 0.83), and 39.4 (AUC 0.84), respectively. In addition, SCB for P-UE, P-Interference, and P-Intensity were 8.1 (AUC 0.78), −10.7 (AUC 0.80), and −11.4 (AUC 0.75), respectively (Table IV). Using the thresholds identified in this study, we then calculated the number of patients who met each outcome measure. For P-UE, 57 patients met the MCID threshold, 58 met the PASS threshold, and 49 met the SCB threshold. For P-Interference, 53 patients achieved MCID, 65 achieved PASS, and 47 achieved SCB. Similarly, for P-Intensity, 46 patients reached MCID, 52 reached PASS, and 41 reached SCB (Table IV).

**Table I:** Demographics of patient sample.

Variable	Mean + SD or no. (%) (n = 72)
Age (yrs)	28.8 (±9.8)
Sex	
Female/male	8 (10.9%)/64 (89.1%)
BMI (kg/m2)	25.8 (±4.7)
ASA class	
1/2/3	43 (60%)/26 (36%)/3 (4%)
Smoking	
Formerly or currently smoking	15 (21%)
Hypertension	6 (8.3%)
Diabetes	0 (0.0%)
Anxiety	9 (12.5%)
Depression	6 (8.3%)
Procedure on dominant arm	38 (52.8%)
Prior traumatic shoulder injury	38 (52.8%)
Seizure disorder	3 (4.2%)
Generalized joint laxity	27 (37.5%)
History of recurrent dislocations	34 (47.2%)
Glenoid bone loss (%)	17 (± 6)
GBL 10%-< 15%	8 (11.1%)
GBL 15%-< 20%	28 (38.9%)
GBL ≥ 20%	36 (50.0%)
Hill-Sachs lesion depth (mm)	4.2 (± 2.3)
Off track	29 (40.3%)
Primary Latarjet	44 (61.1%)
Revision Latarjet∗	28 (38.9%)
Open Latarjet procedure	60 (83.3%)
Arthroscopic Latarjet procedure	12 (16.7%)
Competitive athlete†	23 (31.9%)

*ASA*, American Society of Anesthesiologists; *GBL*, glenoid bone loss; *SD*, standard deviation; *BMI*, body mass index.

∗ Following arthroscopic Bankart repair.

† Defined as participation in organized sports at the high school level or higher, with regular practice or competition within 12 mo prior to surgery.

**Table II:** Preoperative and postoperative PROMIS scores and mean follow-up (mo), stratified by primary vs. revision LP and open vs. arthroscopic technique.

	Full cohort N = 72 (mean ± SD)	Primary LP N = 44 (mean ± SD)	Revision LP N = 28 (mean ± SD)	*P*-value∗	Open LP N = 60 (mean ± SD)	Arthroscopic LP N = 12 (mean ± SD)	*P* value∗
Preoperative PROMIS							
P-UE	38.2 ± 9.5	38.2 ± 9.2	38.7 ± 9.1	.799	37.7 ± 9.4	41.7 ± 6.7	.123
P-Interference	57.4 ± 7.4	57.7 ± 7.4	56.7 ± 7.1	.432	57.6 ± 7.2	55.8 ± 7.6	.506
P-Intensity	46.7 ± 9.1	46.2 ± 8.9	46.9 ± 9.3	.733	46.7 ± 9.3	45.1 ± 8.9	.739
Postoperative PROMIS							
P-UE	51.8 ± 10.1	51.3 ± 9.6	54.0 ± 9.4	.175	54.0 ± 9.4	51.3 ± 9.6	.270
P-Interference	44.1 ± 9.8	45.0 ± 8.2	43.5 ± 7.6	.314	43.5 ± 7.6	45.0 ± 8.2	.180
P-Intensity	35.7 ± 7.9	35.2 ± 6.5	35.3 ± 7.7	.631	35.3 ± 7.7	35.2 ± 6.5	.305
Mean follow-up (mo)	24.2 ± 12.1	25.5 ± 12.2	24.6 ± 10.2	.890	24.6 ± 10.2	25.5 ± 12.2	.140

*LP*, Latarjet procedure; *MCID*, minimal clinically significant difference; *PASS*, patient acceptable symptomatic state; *PROMIS*, patient-reported outcome measurement information system; *P-Intensity*, PROMIS pain intensity; *P-Interference*, PROMIS pain interference; *P-UE*, PROMIS upper extremity; *SCB*, substantial clinicalbenefit; *SD*, standard deviation.

∗ Significance testing performed using Wilcoxon rank-sum test.

**Table III:** Anchor survey responses.

Response option	Anchor pain (n)	Anchor function (n)
Much better	53	53
Better	10	11
Slightly better	3	3
No change	3	3
Slightly worse	1	1
Worse	1	0
Much worse	1	1

Response option	Anchor satisfaction (n)
Very satisfied	50
Satisfied	15
Neither satisfied or dissatisfied	4
Dissatisfied	2
Very dissatisfied	1

**Table IV:** MCID, PASS, and SCB values for PROMIS upper extremity, pain interference, and pain intensity.

	Value	Youden's Index (J)	Accuracy	Sensitivity	Specificity	AUC	Initial PROMIS score	Follow-up PROMIS score	Cohort size (achieved threshold)
P-UE MCID	3.2	0.72	0.86	0.86	0.86	0.88	38.2	51.8	57/72 (79.2%)
P-UE PASS	42.6	0.88	0.89	0.88	1.00	0.92	38.2	51.8	58/72 (80.6%)
P-UE SCB	8.1	0.46	0.76	0.81	0.65	0.78	38.2	51.8	49/72 (68.1%)
P-Intensity MCID	−9.4	0.58	0.72	0.70	0.88	0.84	46.7	35.7	46/72 (63.9%)
P-Intensity PASS	39.4	0.61	0.78	0.77	0.83	0.84	46.7	35.7	52/72 (72.2%)
P-Intensity SCB	−11.4	0.40	0.70	0.69	0.71	0.75	46.7	35.7	41/72 (56.9%)
P-Interference MCID	−6.3	0.69	0.82	0.81	0.88	0.86	57.4	44.1	53/72 (73.6%)
P-Interference PASS	56.7	0.62	0.93	0.95	0.67	0.83	57.4	44.1	65/72 (90.3%)
P-Interference SCB	−10.7	0.59	0.81	0.82	0.76	0.80	57.4	44.1	47/72 (65.3%)

*AUC*, area under curve; *PROMIS*, patient-reported outcome measurement information system; *P-Intensity*, PROMIS pain intensity; *P-Interference*, PROMIS pain interference; *P-UE*, PROMIS upper extremity; ROC, receiver operating curve; *MCID*, minimal clinically significant difference; *PASS*, patient acceptable symptomatic state; *SCB*, substantial clinical benefit.

MCID, PASS, and SCB calculations as determined by ROC curve analysis using Youden index and AUC analysis.

### Factor associated with achieving patient acceptable symptomatic state, minimal clinically important difference, and substantial clinical benefit

For P-UE, generalized joint laxity was associated with lower ORs in achievement of SCB (OR: 0.27, *P* = .04), and male sex was associated with higher OR (OR:8.41, *P* = .04) in achievement of SCB (Table V).

**Table V:** Factors affecting MCID, PASS, and SCB for PROMIS upper extremity function.

	Logistic regression, *P* value		OR (95% CI)
	Univariate	Multivariate	
MCID			
Preoperative P-Interference score	**.005∗**	.130	
Preoperative P-UE score	**.003∗**	.249	
Preoperative P-Intensity score	**.008∗**	.691	
Preoperative external rotation	.089	.266	
Generalized joint laxity	**.049∗**	.060	
ASA 3	.088	.410	
SCB			
Preoperative P- Interference score	**.010∗**	.388	
Preoperative P-UE score	**.006∗**	.284	
Preoperative P-Intensity score	**.011∗**	.492	
Procedure on dominant arm	.151	.745	
Competitive athlete status	.208	.226	
Generalized joint laxity	**.025∗**	**.043∗**	**0.27 (0.07-0.92)**
Male sex	.064	**.037∗**	**8.41 (1.26-76.64)**
PASS			
Hill-Sachs lesion depth	**.042∗**	.273	
Preoperative P-UE score	**.034∗**	.072	
Preoperative external rotation	.086	.066	
Age at surgery (yr)	.182	.544	
Off track Hill-Sachs lesion	.158	.946	
ASA 3	.075	.262	
Male sex	**.032∗**	.055	

*ASA*, American Society of Anesthesiologists; *CI*, confidence interval; *MCID*, minimal clinically significant difference; *OR*, odds ratio; *PASS*, patient acceptable symptomatic state; *PROMIS*, patient-reported outcome measurement information system; *P-Intensity*, PROMIS pain intensity; *P-Interference*, PROMIS pain interference; *P-UE*, PROMIS upper extremity; *SCB*, substantial clinical benefit.

Factors that achieved a *P* value ≤ .20 in their univariate analysis, along with their corresponding *P* values from the subsequent multivariate analysis, are presented. Bold *P* values with asterisks indicate statistical significance.

For P-Interference, history of recurrent dislocations was associated with lower ORs in achievement of MCID (OR: 0.20, *P* = .04). Higher preoperative P-Interference scores were associated with higher ORs in P-Interference SCB achievement (OR: 1.10, *P* = .03). Lastly, higher preoperative P-UE scores were associated with higher ORs in PASS achievement (OR: 1.19, *P* = .03) (Table VI).

**Table VI:** Factors affecting MCID, PASS, and SCB for PROMIS pain interference.

	Logistic regression, *P* value		OR (95% CI)
	Univariate	Multivariate	
MCID			
Preoperative P- Interference score	.057	.125	
Preoperative P-Intensity score	.151	.752	
Preoperative external rotation	.092	.600	
BMI	.058	.371	
Postoperative follow-up duration (mo)	.060	.162	
History of recurrent dislocations	**.035∗**	**.041∗**	**0.20 (0.04-0.87)**
Male sex	.122	.134	
SCB			
Preoperative P-Interference score	**.034∗**	**.033∗**	**1.10 (1.01-1.21)**
BMI	**.026∗**	.216	
Procedure on dominant arm	.167	.429	
Arthroscopic procedure	.166	.263	
Current smoker	.188	.161	
History of recurrent dislocations	.116	.052	
History of anxiety diagnosis	.144	.329	
PASS			
Hill-Sachs lesion depth	.152	.237	
Preoperative P-Interference score	.177	.635	
Preoperative P-UE score	**.008∗**	**.027∗**	**1.19 (1.03-1.42)**
Preoperative P-Intensity score	.115	.844	
Preoperative VAS scores	.073	.450	
History of recurrent dislocations	.195	.166	

*BMI*, body mass index; *CI*, confidence interval; *MCID*, minimal clinically significant difference; *OR*, odds ratio; *PASS*, patient acceptable symptomatic state; *PROMIS*, patient-reported outcome measurement information system; *P-Intensity*, PROMIS pain intensity; *P-Interference*, PROMIS pain interference; *P-UE*, PROMIS upper extremity; *SCB*, substantial clinical benefit; *VAS*, visual analog score.

Factors that achieved a *P* value ≤ .20 in their univariate analysis, along with their corresponding *P* values from the subsequent multivariate analysis, are presented. Bold *P* values with asterisks indicate statistical significance.

For P-Intensity, higher preoperative P-Intensity scores (OR: 1.22, *P* = .003) and being a current smoker (OR: 9.03, *P* = .02) were associated with higher ORs in achievement of MCID. Generalized joint laxity (OR: 0.18, *P* = .02) and history of recurrent dislocations (OR: 0.17, *P* = .02) were associated with lower ORs in achievement of P-Intensity MCID. Larger Hill-Sachs lesion depth was associated with lower ORs in P-Intensity PASS (OR: 0.71, *P* = .04), while higher BMI was associated with higher ORs in P-Intensity PASS achievement (OR: 1.20, *P* = .05) (Table VII).

**Table VII:** Factors affecting MCID, PASS, and SCB for PROMIS pain intensity.

	Logistic regression, *P* value		OR (95% CI)
	Univariate	Multivariate	
MCID			
Preoperative P-Interference score	**.011∗**	.423	
Preoperative P-UE score	**.031∗**	.196	
Preoperative P-Intensity score	**<.001∗**	**.003∗**	**1.22 (1.08-1.42)**
History of recurrent dislocations	.070	**.016∗**	**0.17 (0.03-0.66)**
Current smoker	.155	**.022∗**	**9.03 (1.62-76.32)**
Generalized joint laxity	.103	**.022∗**	**0.18 (0.03-0.71)**
SCB			
Preoperative P-Interference score	**.006∗**	.251	
Preoperative P-UE score	.091	.052	
Preoperative P-Intensity score	**.001∗**	**.003∗**	**1.23 (1.07-1.42)**
Competitive athlete status	.142	.148	
History of recurrent dislocations	.111	**.036∗**	**0.23 (0.07-0.73)**
Generalized joint laxity	**.034∗**	**.014∗**	**0.17 (0.05-0.56)**
PASS			
Hill-Sachs lesion depth	**.040∗**	**.035∗**	**0.71 (0.49-0.96)**
Preoperative P-UE score	**.035∗**	.317	
Preoperative P-Intensity score	.175	.996	
BMI	.050	**.048∗**	**1.20 (1.03-1.47)**
Preoperative VAS score	.106	.888	
History of recurrent dislocations	.065	.099	
Off track Hill-Sachs lesion	.118	.826	
Generalized joint laxity	.178	.066	

*CI*, confidence interval; *MCID*, minimal clinically significant difference; *OR*, odds ratio; *PASS*, patient acceptable symptomatic state; *PROMIS*, patient-reported outcome measurement information system; *P-Intensity*, PROMIS pain intensity; *P-Interference*, PROMIS pain interference; *P-UE*, PROMIS upper extremity; *SCB*, substantial clinical benefit; *VAS*, visual analog score; *BMI*, body mass index.

Factors that achieved a *P* value ≤ .20 in their univariate analysis, along with their corresponding *P* values from the subsequent multivariate analysis, are presented. Bold *P* values with asterisks indicate statistical significance.

For each of the PROMIS instruments, ES was large: UE (1.6, 95% CI:1.5, 1.7), P-Interference (1.4, 95% CI: 1.3, 1.6), and P-Intensity (1.7, 95% CI 1.5, 1.8). Moreover, SRM was determined to be large for each PROM: UE (3.4, 95% CI: 3.2, 3.5), P-Interference (1.2, 95% CI: 1.1, 1.3), and P-Intensity (1.2, 95% CI 1.0, 1.3).

## Discussion

In this study, we have determined the MCID, SCB, and PASS for PROMIS instruments following the LP. Consistent with prior literature on risk factors associated with inferior outcomes following anterior stabilization surgery, we found that generalized joint laxity, history of recurrent dislocations, and Hill-Sachs lesions depth were factors associated with lower propensity for achieving these benchmarks for PROMIS instruments.^17^^,^^39^^,^^50^ Furthermore, we found that male sex, higher BMI, and smoking status were associated with higher odds of achieving the thresholds for SCB for P-UE, PASS for P-intensity, and MCID for P-Intensity, respectively. It is important to note that our study demonstrated excellent responsiveness and reliability (AUC > 0.8) aside from P-UE SCB and P-Intensity SCB, which demonstrated adequate reliability (AUC > 0.7).^62^

Much of the current literature on PROMs emphasizes statistically significant differences, which may not correspond to clinically meaningful improvement as perceived by patients.^11^ Metrics such as the MCID and SCB have been developed to identify the degree of improvement that patients consider noticeable or worthwhile.^11^^,^^13^^,^^15^These values are not fixed and can vary by procedure, pathology, patient population, and calculation methodology.^18^^,^^64^ Pasqualini et al^51^ and Menendez et al^44^ previously reported MCID and PASS values for the Athletic Shoulder Outcome Scoring System, ROWE, Western Ontario Shoulder Instability, American Shoulder and Elbow Surgeons, Single Assessment Numeric Evaluation, and visual analog scale scores in patients undergoing the LP. While Menendez et al used distribution-based methods, Pasqualini employed a hybrid approach, using distribution-based methods to define MCID and anchor-based methods to define PASS. In contrast, the present study employed a fully anchor-based methodology to derive SCB in addition to MCID and PASS values, incorporating patient-reported assessments of improvement and satisfaction to establish thresholds that more closely reflect real-world patient experience.^13^^,^^20^^,^^44^^,^^47^ In addition, to our knowledge, this is the first study to define anchor-based MCID, SCB, and PASS values for PROMIS instruments in the Latarjet population. As PROMIS continues to gain traction because of its adaptability and lower survey burden, these findings offer a practical framework for interpreting postoperative recovery.^59^ Future studies with larger, multicenter cohorts and longer-term follow-up may help validate these thresholds and assess their generalizability across diverse patient populations.

Current literature regarding MCID and SCB related to the Latarjet is exceedingly limited, with no study detailing MCID values for PROMIS instruments. Evaluating underlying factors associated with achieving the MCID, SCB, and PASS is necessary when using PROMIS scores for the assessment of patient satisfaction and outcomes. Higher preoperative PROMIS P-Interference and P-Intensity scores were associated with significantly higher odds of achieving MCID and SCB, suggesting that patients with greater preoperative pain levels and pain-related disruption in daily activities have a higher likelihood of significant postoperative improvement. Similar findings have been reported in studies on shoulder procedures such as arthroscopic rotator cuff repair, where higher preoperative pain levels predicted greater odds of clinically significant improvement.^13^^,^^70^ Comparable trends were observed for smoking and BMI in our analysis: current smokers showed higher odds of achieving P-Intensity MCID, while higher BMI was linked to greater odds of achieving P-Intensity PASS. Smoking, which has been associated with increased wound infections, reoperations, and readmissions following the LP^14^ may also be linked to higher preoperative P-Intensity and P-Interference. The association between smoking and achievement of P-Intensity MCID is intriguing and aligns with prior studies reporting higher baseline pain levels in smokers, which increases the likelihood of achieving MCID postoperatively.^29^ Likewise, existing literature has demonstrated a relationship between BMI and preoperative pain, with improved pain outcomes often seen in patients with higher BMI.^10^^,^^23^^,^^58^ Of note, despite a relatively high mean preoperative P-Interference score (57.4), the PASS threshold for P-interference in our cohort was similarly high (56.7). This may suggest that even modest improvements in P-Interference are acceptable to patients undergoing the LP, particularly when accompanied by substantial gains in range of motion and shoulder function.

Patients with a history of recurrent dislocations had lower odds of achieving MCID for P-Interference and MCID/SCB for P-Intensity. Although the time from initial dislocation to surgery and the specific number of dislocations prior to surgery were not evaluated in this study, recurrent dislocations often suggest prolonged symptom duration, which has been linked to poorer postoperative outcomes.^39^

Male sex was associated with higher odds of achieving P-UE SCB and P-Intensity PASS. Literature on sex-related differences in shoulder instability surgery show mixed results. Some studies have challenged,^9^ while others have endorsed,^49^ male sex as a factor associated with shoulder instability. Prior studies have identified a higher rate of traumatic instability and initial sports-related instability in males compared to females.^38^ Consequently, males may be more likely to return to high-demand physical activities postsurgery, which could influence their perception of substantial benefit when functionality is restored. Ultimately, we believe our findings likely reflect activity-related differences between males and females and not necessarily biology of sex.

Considering bony pathology, increasing Hill-Sachs depth was associated with a lower propensity for achieving P-Intensity PASS (*P* = .04). Interestingly, both the extent of GBL and presence of an off-track Hill-Sachs lesion did not affect achieving MCID, SCB, or PASS values. When evaluating satisfaction following arthroscopic shoulder stabilization, a study of 195 patients by Park et al found a larger width of Hill-Sachs lesion to be associated with higher rates of patient dissatisfaction (regression coefficient: 0.052, *P* = .01).^50^ Overall, the relationship between lesion morphology and patient-reported outcomes in shoulder stabilization procedures appears complex and merits further exploration.

Laxity of the glenohumeral joint is a preoperative factor that may require careful consideration, as laxity differs from instability and is characterized by increased joint translation, greater range of motion, and easier distractibility due to the length and elasticity of stabilizing structures.^26^ In our cohort, patients with generalized joint laxity were significantly less likely to achieve SCB for P-UE (*P* = .04) and MCID (*P* = .02) and SCB (*P* = .01) for P-Intensity. In a study of 420 LPs, Di Giacomo et al found that hyperlaxity—whether congenital or acquired—was associated with a higher risk of postoperative recurrent instability.^17^ Similarly, Voos et al demonstrated that patients with hyperlaxity undergoing ABR had a 3.3-fold higher risk of recurrent instability compared to those without hyperlaxity.^68^ Although our cohort had only 1 case of postoperative dislocation, hyperlaxity may pose detrimental effects to patients undergoing shoulder stabilization procedures. For example, these patients may experience residual laxity in the postoperative period, leading to feelings of instability or limited functional improvement. In addition, while prior studies have suggested an association between female sex and ligamentous laxity,^6^^,^^35^ only 3 of the 27 patients with generalized joint laxity in our cohort were female, and given the small number of female patients overall (n = 8), we were unable to perform an interaction analysis between these variables.

Several limitations exist within our study. First, MCID, SCB, and PASS values can vary with different wording of the anchor questions.^64^ In addition, the collection of PROMIS information telephonically and via the internet can introduce a potential limitation. Although multiple studies have shown this to lead to no significant difference, there is one study that did find a significant difference and so it must be considered a limitation.^4^^,^^7^^,^^31^^,^^69^ Likewise, changing the time to follow-up for postoperative PROMIS collection may yield different MCID values.^64^ The broad age range of our cohort represents a potential limitation; however, the mean age (29 years) was consistent with typical Latarjet populations. The upper range was influenced by a small number of older outliers with high functional demands and persistent instability, for whom a Latarjet was deemed appropriate. Although our cohort of 72 patients met statistical power thresholds, it may not encompass the full spectrum of variability in the outcomes assessed; future studies with larger cohorts may refine our estimates and improve generalizability of our findings. Next, the pooling of both primary and revision LPs (along with open and arthroscopic cases) can introduce heterogeneity in the patient cohort. Differences in symptom duration, degree of GBL, and tissue quality may exist amongst primary shoulder instabilities vs. secondary shoulder instabilities (surgical recurrences) that may influence preoperative and postoperative outcome scores. However, in our cohort, we did not observe any substantial differences in baseline and postoperative outcome scores between primary and revision, or open and arthroscopic cases that warranted separate analyses. Next, our study included patients with a minimum 1-year follow-up, which may not be long enough to thoroughly assess certain long-term surgical complications, such as recurrent instability or posttraumatic osteoarthritis. However, assessing postoperative complications was not a primary focus of our study design. Subsequently, while 12 months was the minimum, the mean follow-up duration in our cohort was approximately 24 months, providing a broader window to assess short-term and mid-term outcomes. Furthermore, studies have indicated that functional improvements following stabilization procedures typically occur within the first year after surgery.^36^^,^^57^ Next, our models did not account for the specific number of dislocations prior to surgery, the presence of chondral lesions, or postoperative coracoid position—factors that may influence patient outcomes. Although we included competitive athlete status in our regression models, we did not stratify these athletes into contact vs. noncontact subgroups. It is important to remember our reported thresholds are in the context of one PROM (PROMIS) and do not provide a universal threshold. Their application within the clinical setting comes with inherent limitations, necessitating an understanding of the patient-physician relationship and the goals of care for the patient.^27^ While PROMIS instruments are validated and increasingly used, we did not compare them to legacy outcome measures (eg, American Shoulder and Elbow Surgeons, Constant, or Western Ontario Shoulder Instability) in this study. However, prior studies on shoulder stabilization procedures have demonstrated moderate-to-strong correlations between PROMIS domains and legacy scores, supporting their validity in this population.^1^^,^^52^ Lastly, PROMIS instruments may not fully capture certain domains of recovery that are particularly relevant in shoulder stabilization patients, such as psychological apprehension, sport-specific function, and activity confidence.

## Conclusion

This study establishes MCID, SCB, and PASS values for PROMIS instruments following the LP. Knowledge of these thresholds and factors associated with achieving them provide surgeons useful tools for predicting and measuring clinically meaningful outcomes following surgery.

## Disclaimer

Funding: No outside funding or grants were received in support of the completion of this study.

Conflicts of interest: The authors, their immediate families, and any research foundation with which they are affiliated have not received any financial payments or other benefits from any commercial entity related to the subject of this article.
